# QM/MM simulations identify the determinants of catalytic activity differences between type II dehydroquinase enzymes†
†Electronic supplementary information (ESI) available: Fig. S1–S5, Tables S1–S3 and extra details on umbrella sampling simulations. See DOI: 10.1039/c8ob00066b


**DOI:** 10.1039/c8ob00066b

**Published:** 2018-05-16

**Authors:** Emilio Lence, Marc W. van der Kamp, Concepción González-Bello, Adrian J. Mulholland

**Affiliations:** a Centre for Computational Chemistry , School of Chemistry , University of Bristol , Cantock's Close , BS8 1TS Bristol , UK . Email: adrian.mulholland@bristol.ac.uk ; Tel: +44 (0)117 9289097; b Centro Singular de Investigación en Química Biolóxica e Materiais Moleculares (CIQUS) , Departamento de Química Orgánica , Universidade de Santiago de Compostela , Jenaro de la Fuente s/n , 15782 Santiago de Compostela , Spain . Email: concepcion.gonzalez.bello@usc.es ; Tel: +34 881 815726; c School of Biochemistry , University of Bristol , University Walk , BS8 1TD Bristol , UK . Email: marc.vanderkamp@bristol.ac.uk ; Tel: +44 (0)117 3312147

## Abstract

Type II dehydroquinase enzymes (DHQ2), recognized targets for antibiotic drug discovery, show significantly different activities dependent on the species: DHQ2 from *Mycobacterium tuberculosis* (*Mt*DHQ2) and *Helicobacter pylori* (*Hp*DHQ2) show a 50-fold difference in catalytic efficiency. Revealing the determinants of this activity difference is important for our understanding of biological catalysis and further offers the potential to contribute to tailoring specificity in drug design. Molecular dynamics simulations using a quantum mechanics/molecular mechanics potential, with correlated *ab initio* single point corrections, identify and quantify the subtle determinants of the experimentally observed difference in efficiency. The rate-determining step involves the formation of an enolate intermediate: more efficient stabilization of the enolate and transition state of the key step in *Mt*DHQ2, mainly by the essential residues Tyr24 and Arg19, makes it more efficient than *Hp*DHQ2. Further, a water molecule, which is absent in *Mt*DHQ2 but involved in generation of the catalytic Tyr22 tyrosinate in *Hp*DHQ2, was found to destabilize both the transition state and the enolate intermediate. The quantification of the contribution of key residues and water molecules in the rate-determining step of the mechanism also leads to improved understanding of higher potencies and specificity of known inhibitors, which should aid ongoing inhibitor design.

## Abstract 2

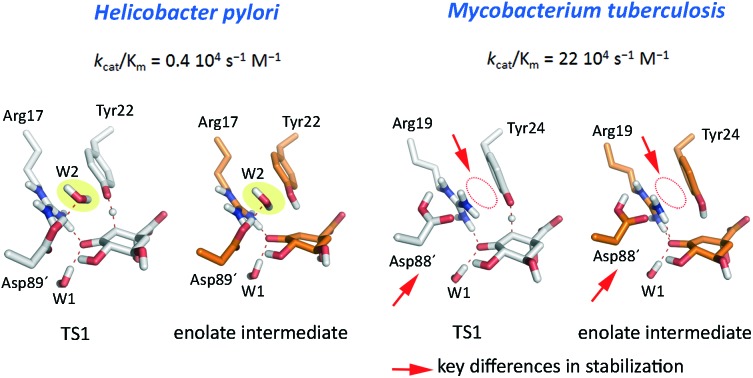
Multiscale simulations pinpoint specific interactions responsible for differences in stabilization of key reacting species in two recognized targets for antibiotic development.

## Introduction

It is well known that the catalytic efficiency of homologous enzymes can vary significantly depending on the species. In most cases the active site of homologous enzymes is highly conserved, and it is generally not obvious how other differences in amino acid sequence may affect activity. Understanding such differences can be used for designing specific inhibitors against enzymes, for example for developing new drug candidates to combat pathogenic bacteria that have developed resistance against existing antibiotics. The emergence and worldwide spread of multidrug-resistant microbial strains is one of the most important challenges for public health in the 21^st^ century.^1^–^3^ The lack of effective antibiotics is currently undermining our ability to deal with infectious diseases, and to manage complications in vulnerable patients undergoing general surgery, organ transplantation, dialysis and chemotherapy for cancer, for which the ability to treat secondary infections is crucial. It has been estimated that if antibiotic resistance continues rising at this rate we will pass from the current 700 000 deaths annually to about 10 million (one every 3 seconds) in 2050.^4^,^5^ Most of the antibiotics in clinical use target the same type of bacterial functions and resistance to them is now widespread and well known. It is therefore not surprising that much effort is currently being devoted to search not only for more effective antibiotics, but also to develop novel chemical entities disabling unexploited essential processes in bacteria. In this context, it is relevant to study the biochemical behavior of those unexplored targets in detail.^1^–^3^,^6^–^8^

A particular goal is to develop more specific antimicrobials, *i.e.* drugs that target particular types of organism, to treat specific infections and diseases. This will require understanding of the essential differences between the same target from different organisms. For enzyme targets, it will be important to understand the differences in catalysis and mechanism. Understanding and analysis of differences in activity and specificity should assist in structure-based design of more specific antimicrobials, targeting specific enzymes. The origins of such differences are generally not obvious from structural data alone: for example, differences in (*e.g.* carbapenemase) activity between various Class A beta-lactamases cannot be discerned from their structures.^9^ However, molecular simulations can identify differences, effectively acting as a ‘computational assay’ of biological activity,^10^ and – crucially – analyze the physical origins of these differences at the molecular level. For example, simulations of reactions with combined quantum mechanics/molecular mechanics (QM/MM) methods,^11^ distinguish Class A beta-lactamases that can efficiently breakdown carabapenem antibiotics from enzymes that cannot.^12^ Modelling of reactions in enzymes with QM/MM methods can identify mechanisms^13^ and provide atomically detailed knowledge about the key interactions of the substrate(s), reaction intermediate(s) and transition state(s). It can further identify specific role(s) of active site amino acids in catalysis^14^ and conformational changes that the enzyme undergoes in a catalytic cycle.^15^ Alongside explaining differences in catalytic efficiency between enzymes, this detailed knowledge potentially provides new perspectives for the structure- and mechanism-based design of enzyme inhibitors.

Here, we present an extensive QM/MM simulation study of the reaction free energy profile of two homologous enzymes of a recognized target for antibiotic drug design, the type II dehydroquinase enzyme (DHQ2). The approach used, providing detailed understanding of catalytic and mechanistic differences, could be used generally for designing specific inhibitors against enzymes present in certain pathogenic bacteria *vs.* non-pathogenic ones, as well as aiding in the rationalization of experimentally observed differences in inhibitor efficacy among homologous enzymes. In particular, the present study compares the DHQ2 from *Mycobacterium tuberculosis* (*Mt*DHQ2) with that from *Helicobacter pylori* (*Hp*DHQ2). We identify, from QM/MM simulations, the key factors that explain the 50-fold catalytic efficiency difference between the two homologous enzymes.

**Scheme 1:**
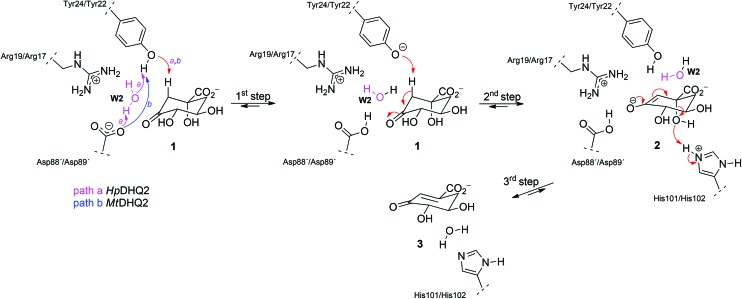
Mechanism of the reaction catalyzed by the *Mt*DHQ2 and *Hp*DHQ2 enzymes. For *Hp*DHQ2 (path a, magenta arrows) the generation of the catalytic tyrosinate during the first step involves a water molecule (**W2**, magenta) whereas for *Mt*DHQ2 (path b, blue arrows) Tyr24 is sufficiently close to Asp88′ to for a direct deprotonation.

**Fig. 1:**
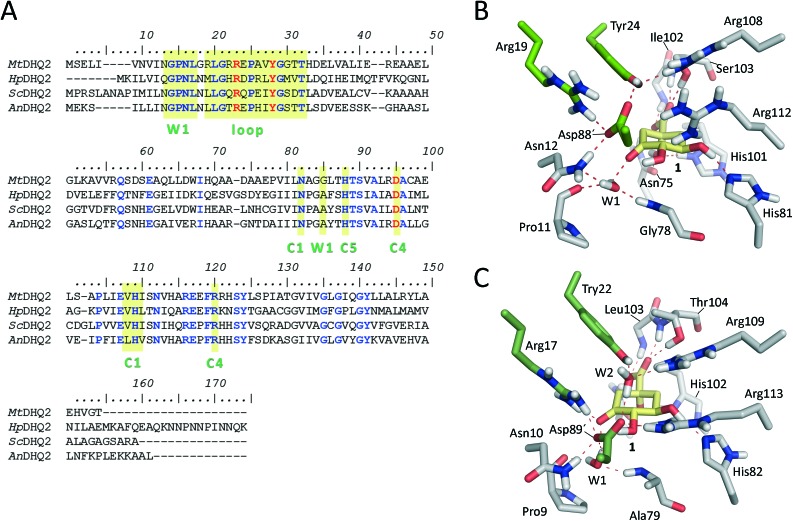
(A) Amino acid sequence alignments for the *M. tuberculosis*, *H. pylori*, *S. coelicolor*, and *A. nidulans* DHQ2 enzymes. Protein sequences were aligned using the CLUSTAL Omega multiple sequence alignment (; http://www.ebi.ac.uk/Tools/msa/clustalo/, accessed July 1, 2017). Conserved and essential residues are highlighted in blue and red, respectively. The substrate-covering loop and the residues involved in the recognition of C1, C4 and C5 positions of the natural substrate and the W1 pocket are shaded with a yellow box. (B, C) Detailed views of the active site residues in Michaelis complex models for *Mt*DHQ2 (B) and *Hp*DHQ2 (C). Relevant residues are shown and labeled. Catalytic residues are indicated with green carbons, substrate with yellow carbons. Hydrogen bonding interactions are indicated as red dashed lines.

DHQ2 (3-dehydroquinate dehydratase, EC 4.2.1.10) is the third enzyme of the shikimic acid pathway through which erythrose-4-phosphate and phosphoenol pyruvate are converted into chorismic acid. The latter is the precursor of important aromatic metabolites such as the aromatic amino acids, folate cofactors, ubiquinone and vitamins E and K.^16^ DHQ2, which is encoded by the *aroD*/*aroQ* gene and does not have any counterpart in human cells, is an essential enzyme for *Mycobacterium tuberculosis*, which causes tuberculosis, as well as for *Helicobacter pylori*, which is a major cause of gastric and duodenal ulcers and has been classified as a class I carcinogen.^17^,^18^ The enzyme is also present in the non-pathogenic bacterium *Streptomyces coelicolor*,^19^ as well as the fungi *Aspergillus nidulans*,^20^ and *Neurospora crassa*.^21^ DHQ2 is a dodecamer (tetramer of trimers), with a trimer as the minimum catalytic unit.^22^ It catalyzes the reversible dehydration of 3-dehydroquinic acid (**1**) to form 3-dehydroshikimic acid (**3**) (Scheme 1). The enzymatic mechanism consists of an overall *anti* elimination of water involving the loss of the more acidic pro-*S* hydrogen from C2 in **1***via* the enolate intermediate **2**.^23^–^25^ Three residues have been identified by chemical modification and site-directed mutagenesis studies as being essential for enzyme activity. Two of them are located in a flexible loop (henceforth the substrate-covering loop) that forms a lid that closes the active site for catalysis: an arginine^26^ (Arg19/Arg17 in *Mt*DHQ2 and *Hp*DHQ2, respectively; this order will be used throughout) and a tyrosine^27^ (Tyr24/Tyr22).^28^ The third residue is an aspartate (Asp88′/Asp89′) from the neighboring enzyme subunit (residues from this subunit will be marked with an accent mark).^25^ The resolution of several crystal structures of DHQ2 in complex with the product and some reversible competitive inhibitors, in combination with diverse biochemical and computational studies allows a detailed description of the active site and provides a good overall knowledge of the enzymatic mechanism. The substrate is anchored to the active site by several hydrogen bonding interactions through the carboxylate group and the hydroxyl groups (Fig. 1B and C). Specifically, the carboxylate group, which is key for recognition, establishes four strong hydrogen bonding interactions involving the main chain amide NH groups of Leu102/Ile103 and Ser103/Thr104, the side chain hydroxyl group of Ser103/Thr104, and the side chain amide of the conserved Asn75/Asn76.^28^ The latter also forms a second hydrogen bond between its carbonyl group and the C1 hydroxyl group, thereby positioning the hydroxyl group to accept a proton from the conserved His101/His102. The main hydrogen bonding interactions of the secondary hydroxyl groups are with His81/His82 and Arg112/Arg113 (C5 hydroxyl group), and Asp88′/Asp89′ (C4 hydroxyl group). In addition, the C3 carbonyl group is anchored to the active site through a structural water molecule (henceforth **W1**) that also hydrogen bonds with residues Pro11/Pro9, Asn12/Asn10 and Gly78/Ala79. Previous computational studies involving QM/MM Steered Molecular Dynamics (SMD) simulations of the reaction showed that the reaction is initiated by the essential aspartate (Asp88′/Asp89′).^25^ This residue acts as the general base to deprotonate the essential tyrosine to the catalytic tyrosinate form, which triggers the enzyme-catalyzed chemical reaction. For the *Hp*DHQ2 enzyme, the deprotonation occurs with the assistance of a water molecule (henceforth **W2**), while for *Mt*DHQ2, the tyrosine is directly deprotonated by the aspartate residue. The latter is supported by results from solvent isotope effects and proton inventory studies.^25^ The necessary reduction in p*K*_a_ of Tyr24/Tyr22 has been proposed to be achieved by the proximity of two conserved arginine residues, Arg108/Arg109 and Arg112/Arg113 and a cation–π interaction with the essential Arg19/Arg17.^29^ MD simulation studies revealed that the latter residues are also responsible for the release of the product from the active site.^25^ Importantly, the essential arginine controls the correct conformation of the tyrosinate for the removal of the pro-*S* hydrogen from C2 in **1**. The final step is the acid-catalyzed elimination of the C1 hydroxyl group that is mediated by the conserved histidine His101/His102 acting as a proton donor.

The previously reported computational studies on the mechanism of DHQ2 either included a small model of the active site (lacking the essential arginine or a detailed description of how the catalytic tyrosinate is generated)^24^ or used limited sampling of conformations on the reaction pathway using QM/MM SMD^25^ studies. However, the over 50-fold catalytic efficiency difference between the *M. tuberculosis* (*k*_cat_/*K*_m_ = 22 × 10^4^ s^–1^ M^–1^)^30^ and *H. pylori* (*k*_cat_/*K*_m_ = 0.4 × 10^4^ s^–1^ M^–1^)^30^ enzymes could not be explained; the active site of the DHQ2 enzymes is highly conserved (Fig. 1A).

Here, we present an extensive semiempirical QM/MM Umbrella Sampling simulation study, with corrections up to the correlated *ab initio* (MP2) level, to explore the reaction free energy profile of these two homologous enzymes. Detailed analysis of the key rate-determining step, which involves the formation of the enolate intermediate **2**, allowed us to identify relevant differences in this step that explain the experimentally observed difference in catalytic efficiency of the two studied enzymes, *Mt*DHQ2 and *Hp*DHQ2. The results shown here give a detailed picture of the mechanism and identify relevant interactions that could be used for future inhibitor design.

## Results and discussion

**Fig. 2:**
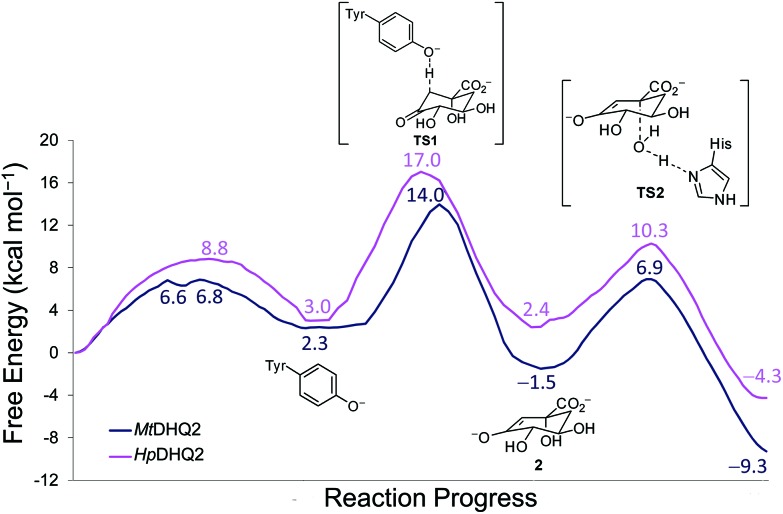
Free energy profiles obtained using Umbrella Sampling simulations at the SCC-DFTB/ff03 level for the whole reaction in both DHQ2 enzymes [*Mt*DHQ2 (blue), *Hp*DHQ2 (magenta)]. For the 2^nd^ step, free energies were corrected using single point MP2/6-31+G(d,p) calculations. For the 1^st^ step in *Hp*DHQ2 and the 3^rd^ step in both enzymes, minimum free energy paths extracted from 2D free energy surfaces are shown.

QM/MM (SCC-DFTB/ff03) umbrella sampling simulations were performed for the two DHQ2 enzymes, *Mt*DHQ2 and *Hp*DHQ2, and for the three reaction steps: (a) generation of the catalytic tyrosinate, (b) formation of the enolate intermediate **2**, and (c) enolate dehydration (Scheme 1). In general, the resulting free energy profiles at this level of theory indicate that the three-step mechanism is feasible, with an overall energy barrier of 15.4 and 16.0 kcal mol^–1^ for *Mt*DHQ2 and *Hp*DHQ2, respectively. The free energy profiles at the SCC-DFTB/ff03 level suggest that the enolate dehydration (3^rd^ step) is the rate-determining step (Fig. S1†), whereas experimental isotope effects indicate that this is due to the formation of the enolate intermediate **2** (2^nd^ step).^23^ Small model calculations indicated that this discrepancy is likely due to inaccuracies of SCC-DFTB for the reaction (and activation) energy of the 2^nd^ step, *i.e.* due to inaccurate proton affinities. Our results show that SCC-DFTB underestimates the stability of the enolate intermediate **2** with respect to the substrate (with tyrosinate) by between 6–8 kcal mol^–1^, when compared with more accurate methods such as MP2 and B3LYP (Fig. S2†). Thus, corrections at the MP2/6-31+G(d,p) level to the energy profile for the 2^nd^ step, give free energy profiles for the three step mechanism with overall barriers of 14.0 and 17.0 kcal mol^–1^ for *Mt*DHQ2 and *Hp*DHQ2, respectively (Fig. 2). These energy values correlate reasonably well with values derived from the experimentally determined apparent catalytic rates (using the Arrhenius equation with a unity pre-factor): 16.5 and 17.4 kcal mol^–1^ for *Mt*DHQ2 and *Hp*DHQ2, respectively. Notably, the 2^nd^ step is now rate-determining for both enzymes, in agreement with the experimental data. A more detailed description of the corrections carried out, as well as the main differences between the two enzymes along the reaction path identified in the herein reported QM/MM (SCC-DFTB/ff03) umbrella sampling simulations is provided below.

### 1st step: Generation of the catalytic tyrosinate

**Fig. 3:**
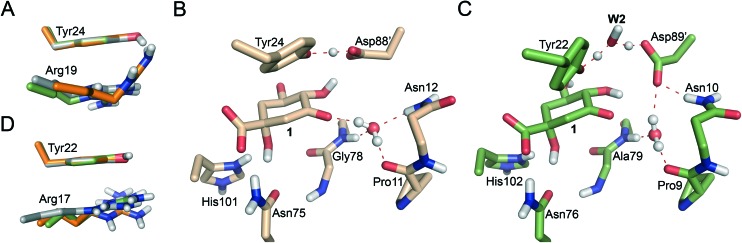
(A) & (D) Comparison of the position of Tyr24/Tyr22 and Arg19/Arg17 residues in the reactants (green), transition state (gray) and products (orange) of the 1^st^ step of the reaction in *Mt*DHQ2 (A) and *Hp*DHQ2 (D). (B) & (C) Representative geometries of the transition state for the formation of the catalytic tyrosinate (1^st^ step) in QM/MM umbrella sampling simulations for *Mt*DHQ2 (B) and *Hp*DHQ2 (C). Relevant residues are shown and labeled. Key hydrogen bonding interactions and bonds broken/formed are indicated as dashed lines.

**Fig. 4:**
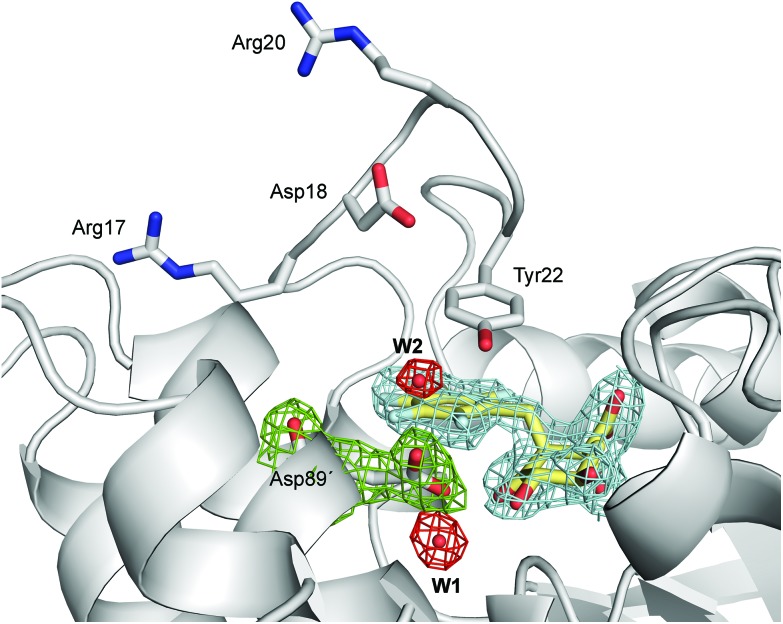
Unbiased electron density for (2*S*)-2-perfluorobenzyl-3-dehydroquinic acid (yellow, inhibitor), **W1**, **W2** and Asp89′ in the binary *Hp*DHQ2/inhibitor complex (chains A and C, PDB code ; 4B6R,^31^ 1.9 Å). A maximum-likelihood weighted 2*F*_o_ – *F*_c_ map contoured at 1*σ* is shown up to 1.6 Å around the inhibitor molecule (blue), **W1** and **W2** (red) and Asp89′ (green).

Reaction barriers of 6.8 and 8.8 kcal mol^–1^ for *Mt*DHQ2 and *Hp*DHQ2, respectively, were found for this step (Fig. 2). The ∼2 kcal mol^–1^ difference between the enzymes can be explained by the distinct nature of the process (Fig. 3). While for *Mt*DHQ2 the essential Tyr24 is deprotonated directly by the essential Asp88′, for *Hp*DHQ2, the process is mediated by a water molecule (**W2**), which is located between Tyr22 and Asp89′. **W2** is observed in diverse crystal structures of binary *Hp*DHQ2/inhibitor complexes such as PDB ; 4B6R (Fig. 4).^31^ It seems that **W2** supplements the inability of the essential Tyr22 to be located close enough to the catalytic Asp89′ for direct proton transfer. This is mainly due to the salt bridge present during the whole reaction between Asp18 and Arg20, both located in the substrate-covering loop, which controls and limits active site flexibility. This strong interaction, which is not present in *Mt*DHQ2 (Fig. 1A, Glu20 and Ala22 are present in equivalent positions), has been proposed to control and reduce the plasticity of this loop (Fig. S2†).^31^ In addition, the presence of Glu20 in the loop of *Mt*DHQ2, replacing the shorter Asp18 in *Hp*DHQ2, favours the interaction between Glu20 and the neutral Asp88′. In fact, we observed that after performing the Umbrella Sampling simulation for the 2^nd^ step (forming the substrate **1**), this interaction leads to a large distance between Tyr24 and the neutral Asp88′ (average distance of 5.1 Å between the Tyr24 hydroxyl O and the Asp88′ carboxylic acid H). To avoid this issue, we introduced an additional reaction coordinate (and performed QM/MM umbrella sampling along this coordinate) to reduce the distance between Tyr24 and Asp88′ and to break the interaction between Glu20 and neutral Asp88′.

Significant differences in the behaviour of the structural water molecule **W1** for both enzymes were identified. While for the *Mt*DHQ2 enzyme, the four hydrogen bonding interactions that anchor **W1** to the active site (also observed in the Michaelis complex and involving residues Pro11, Gly78 and Asn12 and the C3 ketone in **1**) remained unchanged during the whole mechanism, this is not the case for *Hp*DHQ2 (Fig. 3B *vs.* C). For *Hp*DHQ2, only the interactions involving residues Pro9 and Ala79 remained present during the whole mechanism since Asn10 forms a hydrogen bond with Asp89′ (Fig. 3C). As a consequence, **W1** rotates during the 2^nd^ step to form a hydrogen bond with the O3 atom of the forming enolate, an equivalent interaction as observed for the *Mt*DHQ2 enzyme.

Overall, the reaction energy for the 1^st^ step is almost identical (0.7 kcal mol^–1^ difference, Fig. 2). The capability of both enzymes for stabilization of the catalytic tyrosinate is therefore similar. For both enzymes, the formation of the catalytic tyrosinate occurs with a parallel arrangement between the phenol group of the essential tyrosine and the guanidinium group of the essential arginine Arg19/Arg17 (Fig. 3A and D). This arrangement helps to maximize the cation–π interaction between both residues, which has been proposed to (a) control the appropriate position of the tyrosinate for abstraction of the pro-*S* hydrogen atom from C2 in **1**; and (b) to reduce the p*K*_a_ of the tyrosine, along with the conserved Arg108/Arg109 residue.^29^ The latter residue is likely to set up the catalytic tyrosine or **W2** (in *Mt*DHQ2 and *Hp*DHQ2, respectively), for deprotonation. Once the tyrosinate was formed, this parallel arrangement between Tyr24/Tyr22 and Arg19/Arg17 was partially lost, although both residues remain close (Fig. 3A and D).

### 2nd step: Enolate formation

As aforementioned, simulations of the full reaction with SCC-DFTB fail to predict the 2^nd^ step as the partial rate determining step of the reaction path, due to the overestimation of the energy for hydrogen abstraction, as well as the underestimation of the reaction barrier by SCC-DFTB (Fig. S3†); such limitations are typical of this efficient but approximate density-functional theory method (with the recent third-order parametrization showing promising improvement).^32^ To overcome this issue, several high-level QM methods (*ab initio* MP2, and DFT: B3LYP and MPW1K) were employed to correct the free energy profile of this 2^nd^ step, using energy calculations on SCC-DFTB/MM potential energy profiles. All showed the same trends, so only the *ab initio* MP2 results are discussed here (Fig. S4†). Moreover, these QM methods also provide an energy profile for this step with the typical proton-transfer curve, which was not observed using SCC-DFTB. These corrections not only allow the correct identification of the partially rate-determining 2^nd^ step, but also serve to assess the nature of the process and the difference between the two homologous enzymes. Once corrected, the formation of **2** is thermodynamically favorable for both enzymes, by –3.8 and –0.6 kcal mol^–1^ (for *Mt*DHQ2 and *Hp*DHQ2, respectively). As a result, *Mt*DHQ2 stabilizes the enolate intermediate **2** more effectively than *Hp*DHQ2, with a relative difference compared to the Michaelis complex of 3.9 kcal mol^–1^. Further, the energy barrier for the transition state (henceforth **TS1**) in *Mt*DHQ2 is lower than for *Hp*DHQ2 (11.7 *vs.* 14.0 kcal mol^–1^).

**Fig. 5:**
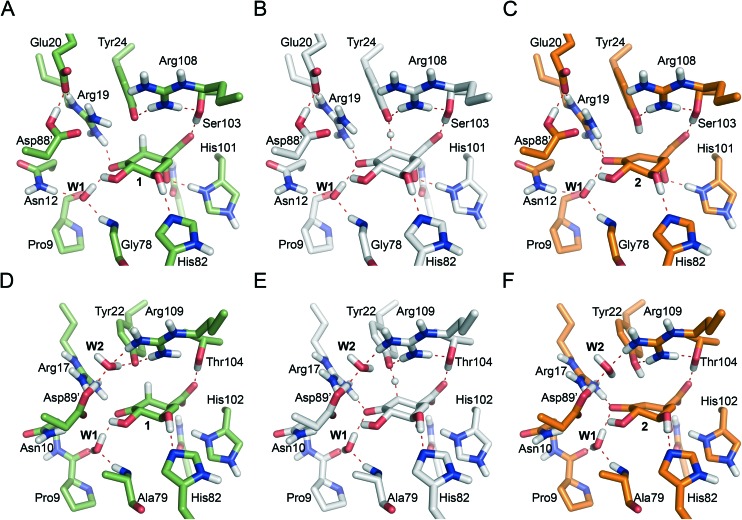
Representative geometries of the catalytic tyrosinate (A & D), **TS1** (B & E) and enolate intermediate **2** (C & F) for the 2^nd^ step of the *Mt*DHQ2 (A–C) and *Hp*DHQ2 (D–F) mechanism. Geometries were taken from the potential energy surface. Relevant residues and water molecules are shown and labeled. Key hydrogen bonding interactions and bonds broken/formed are indicated as red dashed lines.

We evaluate the electrostatic contributions to stabilisation of enolate intermediate **2** and **TS1** of key residues (Arg19/Arg17 and Asp88′/Asp89′) and the structural and catalytic water molecules **W1** and **W2** (Tables 1, S1 and S3†). Significant differences between the two enzymes are identified for each (Fig. 5).

**Table 1:** Contributions of Arg19/Arg17 and Asp88′/Arg89′ and water molecules **W1** and **W2** to the stabilization of **TS1** and the enolate intermediate **2** relative to substrate and tyrosinate state (kcal mol^–1^)^*a*^

Residue/water molecule	**TS1**		Enolate intermediate **2**	
	*Mt*DHQ2	*Hp*DHQ2	*Mt*DHQ2	*Hp*DHQ2
Arg19/Arg17	–2.74	4.30	6.20	15.21
Asp88′/Asp89′	1.51	–2.07	1.67	–4.56
**W1**	1.13	0.89	4.03	2.89
**W2**	n/a	–8.60	n/a	–16.89

^*a*^Energy values were obtained from single point MP2/6-31+G(d,p) calculations and as a difference of the relative energy (with respect to the reactants) of the QM region without the residue minus the energy of the full QM region. Positive values indicate stabilization with respect to the reactants (substrate and tyrosinate state).

#### Arg19/Arg17

**Fig. 6:**
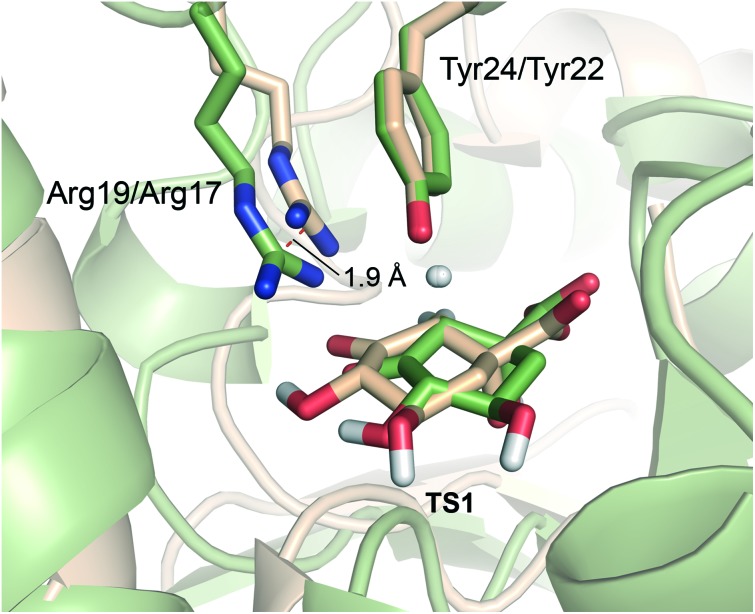
Comparison of **TS1** in the *Mt*DHQ2 (colored as wheat) and *Hp*DHQ2 (green) mechanism. Note that while the arrangement and position of Tyr24/Try22 is quite similar for both enzymes, this is no longer the case for Arg19/Arg17. For *Hp*DHQ2, the essential Arg17 is displaced by about 1.9 Å.

For both enzymes this is the main residue stabilizing the enolate intermediate **2**. Blomberg *et al.* did not include the equivalent Arg23 in their calculations of DHQ2 from *S. coelicolor*,^24^ thus predicting an endoergic process for enolate formation (with energy values ranging between 11.3–14.5 kcal mol^–1^). This confirms the important role of this essential Arg in the stabilization of **2** in DHQ2 enzymes. However, the contribution of this residue to stabilization of **TS1** was found to be different for *Mt*DHQ2 and *Hp*DHQ2. In *Hp*DHQ2, this residue strongly stabilizes **TS1**, whereas this is not the case in *Mt*DHQ2. A key structural difference is that in *Hp*DHQ2, Arg17 is in close (hydrogen bonding) contact with the ketone group of the substrate (during formation of **TS1**), whereas in *Mt*DHQ2 interaction of the guanidinium group of Arg19 with the substrate is rather distant in **TS1** (Fig. 6). Thus, for *Hp*DHQ2, the essential arginine is mainly interacting with the ketone group, whereas in *Mt*DHQ2 it is mostly with the essential tyrosine. Average distances between the NH1, NH2 and CZ atoms in Arg19/Arg17 and the O3 atom in **TS1** in the umbrella sampling window for **TS1** revealed similar values for both enzymes (3.3, 2.9 and 3.5 Å for *Hp*DHQ2 and 2.9, 3.2 and 3.5 Å for *Mt*DHQ2). However, the average distance between the centre of masses of the guanidinium group in Arg19/Arg17 and the phenol group in Tyr24/Try22 is somewhat different (3.5 Å for *Mt*DHQ2 and 4.4 Å for *Hp*DHQ2). Taken together, this shows that (a) the cation–π interaction between Arg19 and Tyr24 appears stronger for *Mt*DHQ2 than for *Hp*DHQ2, (b) in *Mt*DHQ2, Arg19 is exclusively involved in the stabilization of the enolate intermediate **2** and positioning of the catalytic tyrosine for triggering the reaction, and (c) in *Hp*DHQ2, Arg17 has the additional role of activating/positioning the substrate for enolate formation. The distinct role and arrangement of the essential tyrosine for catalysis as well as the enolate stabilization in both enzymes can explain the marked inhibitory potency differences of previously reported reversible competitive inhibitors based on the natural substrate and the enolate intermediate. For *Hp*DHQ2, compounds in which the pro-*S* hydrogen of C2 in **1** was replaced by diverse benzyl groups, compounds **4**, have *K*_i_ values from 1.4 to 0.9 μM, whereas their inhibitory potency against *Mt*DHQ2 increases up to 25-fold (56 to 100 nM) (Table 2).^33^ These inhibitors, which reduce the plasticity of the substrate-covering loop avoiding the cation–π interaction between Tyr24/Tyr22 and Arg19/Arg17 required for catalysis, are more potent against *Mt*DHQ2 because the flexibility of its loop needs to be sufficiently large to locate Tyr24 close enough to Asp89′ for deprotonation. Further, 3-*O*-alkylaryl mimics of the enolate intermediate **2**, compounds **5**, are more potent against *Mt*DHQ2 than against *Hp*DHQ2, which is related to the more effective stabilization of **2** in *Mt*DHQ2 shown here.^34^,^35^

**Table 2:** *K*_i_ (nM) of selected examples of reversible competitive inhibitors of *Mt*DHQ2 and *Hp*DHQ2 enzymes

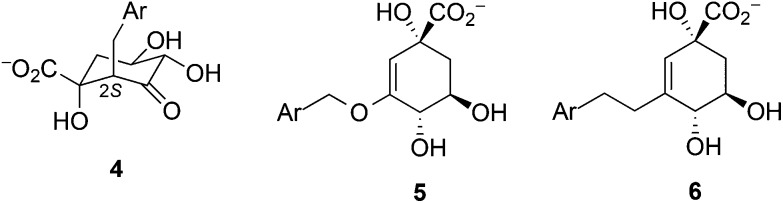				
Compd	Ar	*Mt*DHQ2	*Hp*DHQ2	Ref.
**4a**	(4-OMe)C_6_H_4_	100	1420	33
**4b**	Benzo[*b*]thiophen-5-yl	56	900	33
**4c**	C_6_F_5_	74	970	33
**5a**	Benzo[*b*]thiophen-5-yl	28	132	34 and 35
**5b**	Naphth-2-yl	35	310	34 and 35
**6a**	Benzo[*b*]thiophen-5-yl	254	2460	36
**6b**	Naphth-2-yl	436	790	36

#### Asp88′/Asp89′

While for *Mt*DHQ2, Asp88′ contributes favorably to the stabilization of both the enolate intermediate **2** and **TS1**, the opposite effect is observed for Asp89′ in *Hp*DHQ2. This can be explained from the distinct orientation adopted by the neutral Asp88′/Asp89′ side chain and the effects that this arrangement causes in the nearby residues. In *Mt*DHQ2, the proton of the neutral Asp88′ hydrogen bonds to the side chain of Glu20 (thus keeping Asp88′ away from where the enolization reaction takes place). In contrast, Asp89′ hydrogen bonds to the catalytic water molecule **W2** in *Hp*DHQ2, placing the carbonyl oxygen atom of Asp89′ close to the developing negative charge of enolate **2** O3 (Fig. S5†).

#### Structural water molecule **W1**

W1 has a favorable contribution to stabilization of both **TS1** and the enolate **2** in both enzymes (Table 1). This effect can be explained by the hydrogen bond between **W1** and the O3 oxygen atom of the forming enolate found in both cases, which helps to stabilize the developing negative charge on this atom. The importance of this water molecule for inhibitor binding to DHQ2 has been previously demonstrated: replacing the oxygen atom in the side chain of 3-*O*-alkylaryl mimics of the enolate intermediate **2**, compounds **5**, by a carbon atom (3-alkylaryl derivatives, compounds **6**) leads to a decrease in the inhibitory potency up to 20-fold (Table 2).^34^–^36^ MD simulations of both enzyme/inhibitor complexes showed that this substitution causes an increase in the distance between the oxygen atom of the side chain inhibitors and **W1** of about 1 Å, resulting in the loss of a favorable polar interaction.^37^ In addition, a Comparative Binding Energy (COMBINE) analysis carried out with more than 50 competitive reversible inhibitors of both DHQ2 enzymes revealed that the interaction of the inhibitors with **W1** is the most significant contribution to the inhibitory potency.^37^

#### Catalytic water molecule **W2**

The significant destabilization of both **TS1** and the enolate intermediate **2** by **W2** (only present in *Hp*DHQ2) is surprising (Table 1). To ascertain if this result could be due to a strained arrangement of **W2** in the potential energy surface conformations, the electrostatic interactions of **W2** were also calculated for 50 snapshots from the umbrella sampling windows of the reactant, transition state and product of the 2^nd^ step. No minimization was performed on any of these complexes. Average values of –8.6 ± 0.5 and –16.9 ± 0.3 kcal mol^–1^ (at the SCC-DFTB level of theory) were obtained for **TS1** and **2**, respectively (relative to the reactant state), revealing no significant difference to those obtained from the potential energy surface (–8.0 and –14.6 kcal mol^–1^; Table S1†). Even if only the tyrosinate anion and the substrate were included in the energy calculation (either in presence or absence of **W2**) significantly negative values for **TS1** and **2** (–5.9 and –11.6 kcal mol^–1^, respectively) were obtained. Single point calculations of the strength of hydrogen bonds between **W2** and the catalytic tyrosinate and the enolate **2** using the geometries from the potential energy surface, show that the hydrogen bond with the catalytic tyrosinate is much stronger than that with the enolate **2** (Table S3†). This is in line with the hydrogen bond distances (distance between the **W2** hydrogen and the O3 oxygen atom in **2** is 2.7 Å in the potential energy surface structure and 2.2 Å (average value) in the ensemble of the QM/MM simulation for the products, whereas the **W2** hydrogen to tyrosinate oxygen distances are 1.66 Å and 1.81 Å, respectively). The position of **W2** is not affected significantly by the restraints used to prevent **W2** exchange with a water molecule from the MM region: a 50 ps QM/MM MD simulation of the product state without the relevant restraints, showed that after 26–27 ps, **W2** leaves its position and was almost immediately replaced by another water molecule; in other words, the simulations show this position to be almost always occupied by a water molecule, but some exchange of the water molecule at this site occurs on the MD timescale. Before and after the exchange, strong hydrogen bonding interactions with Arg109 are present, but not with enolate **2** (Fig. S6†). Taken together, the results of these studies indicate that the water molecule **W2** is mainly responsible of the lower catalytic efficiency of the *H. pylori* enzyme: the use of a water molecule for the generation of the catalytic tyrosinate, necessary due to the reduced flexibility of its substrate-covering loop, causes less favorable interactions at the rate-determining step of the enzymatic conversion, resulting in a lower catalytic rate.

### 3rd step: Enolate dehydration

**Fig. 7:**
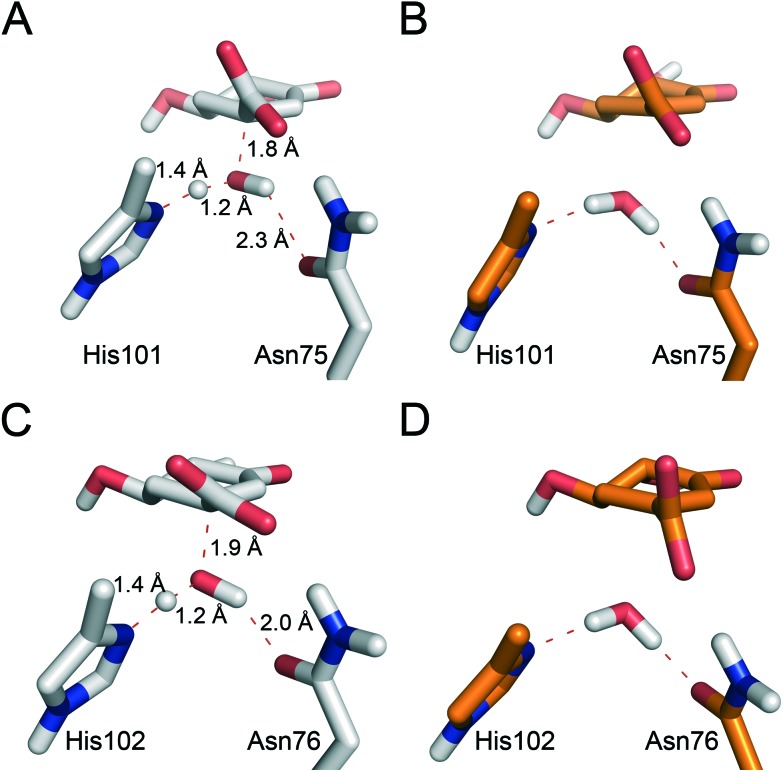
Representative geometries of **TS2** (A & C) and products (B & D) for the 3^rd^ step of the *Mt*DHQ2 (A–B) and *Hp*DHQ2 (C–D) mechanism. Geometries taken from the potential energy surface. Relevant residues and water molecules are shown and labeled. Key hydrogen bonding interactions and bonds broken/formed are indicated as red dashed lines.

No significant differences between the two enzymes were identified for this step either in energy or geometry (Fig. 7). Similar free energy barriers (8.4 and 7.9 kcal mol^–1^) and reaction energies (–7.8 and –6.7 kcal mol^–1^) were obtained for *Mt*DHQ2 and *Hp*DHQ2, respectively. In contrast, Blomberg *et al.*^24^ reported a great variability for the energies barriers calculated for this step and for the energy of the whole process for the *Streptomyces coelicolor* enzyme. In particular, the energy barrier for the two step mechanism (enolate formation and enolate dehydration) found previously with DFT ranged between 14.4 and 22.5 kcal mol^–1^ and showing a maximum energy for the transition state of the enolate dehydration (**TS2**), and the energy of the transformation ranged between –8.2 to 9.2 kcal mol^–1^. Our model is more consistent with the experimental data, with a barrier located in the **TS1** for the formation of the enolate **2**, and an overall energy of –9.3 and –4.3 kcal mol^–1^ (for *Mt*DHQ2 and *Hp*DQH2, respectively) for the three step mechanism.

Taken together, the calculations reported herein allow the quantification of small differences in the energy barriers of catalytic processes carried by homologous enzymes, which is mainly related to *k*_cat_. The differences in recognition (*K*_m_) between both enzymes, which in this case are also experimentally significant and have an important impact on the overall process catalyzed by the enzyme (*k*_cat_/*K*_m_), cannot be quantified by this type of computational study. However, the knowledge in atomic detail of the geometry of the Michaelis complex, transition states and intermediates of the catalytic process in both enzymes and more importantly, the relevant differences found between the mechanisms of both enzymes in terms of interactions between the enzyme and reacting species provides a good understanding of the experimentally observed differences in efficiency [*M. tuberculosis* (fast) *vs. H. pylori* (slow)].

## Conclusions

In the present study we have shown that a combined approach using QM/MM umbrella sampling simulations at the SCC-DFTB/ff03 level of theory with MP2/6-31+G(d,p) corrections on the key reaction step of the reaction catalyzed by the DHQ2 enzyme provides free energy profiles in agreement with experimental data. No significant energetic differences were found for the generation of the catalytic tyrosinate and the enolate dehydration steps in the *Mt*DHQ2 and *Hp*DHQ2 homologues. The 2^nd^ step is rate-determining, in line with experiment (and in contrast with previously reported computational studies), and it is this step which gives rise to observed differences in activity between the two enzymes. Quantification of the contribution of relevant residues in the rate-determining step allowed the identification of the key factors responsible for the 50-fold catalytic efficiency difference between two homologous enzymes, *Mt*DHQ2 and *Hp*DHQ2. The more efficient stabilization of the enolate intermediate by the *M. tuberculosis* enzyme results in a thermodynamically more favorable 2^nd^ step (Δ*G* –3.8 kcal mol^–1^) than for its *H. pylori* homologue (–0.6 kcal mol^–1^). For *Hp*DHQ2, a conserved water molecule (**W2**) between the catalytic residues Asp89′ and Tyr22 was found to destabilize both the transition state (**TS1**) and the enolate product **2** of the 2^nd^ step. The water molecule **W2** that is required for the 1^st^ step in *Hp*DHQ2 (probably to compensate for the inability to locate both residues close enough for the generation of the catalytic tyrosinate) leads to a less optimal arrangement of the active site for the subsequent steps of the enzymatic conversion. The presence of **W2** (and the lack of the Asp–Glu interaction observed in *Mt*DHQ2) also contributes to the destabilising influence of Asp89′ (compared to stabilization by Asp88′ in *Mt*DHQ2). The requirement for **W2** in *Hp*DHQ2 is probably determined by the more limited flexibility of the substrate-covering loop in this enzyme, due to the formation of an internal salt bridge between two residues of this loop, Asp18 and Arg20. Comparison of the amino acid sequence of the catalytic loop in diverse DHQ2 homologous enzymes reveals that *Mt*DHQ2 is the only one with an apolar residue in one of the corresponding positions (Ala22; Fig. 1A), which appears to be crucial for its larger flexibility.^31^

Our quantification of the contributions of the key residues in the rate-determining step of the mechanism provides a better understanding of the higher inhibitory potencies of known mechanism-based inhibitors, *i.e.* analogs of the natural substrate and the enolate intermediate. Whereas the crystal structures of the binary DHQ2/ligand complexes for both enzymes show a highly similar binding mode (*e.g.* PDB codes ; 4B6O 31 and ; 2Y71 35 for *Mt*DHQ2 and PDB codes ; 4B6R 31 and ; 2WKS 34 for *Hp*DHQ2), the inhibitors present in these structures have significantly distinct inhibitory potencies. The identified more efficient stabilization of the enolate intermediate and **TS1** by the *M. tuberculosis* enzyme explains the difference in inhibition data and will be helpful for ongoing inhibitor design. The computational studies presented here highlight the potential of QM/MM simulations as a tool for understanding differences in catalytic efficiency between homologous enzymes.

## Experimental

### System preparation

Initial structures were prepared as in ref. 25. For *Mt*DHQ2, the coordinates from the crystal structure of the *M. tuberculosis* enzyme in complex with the product, 3-dehydroshikimic acid (**3**) (Protein Databank (PDB) code ; 3N59),^38^ were used. For *Hp*DHQ2, the crystal structure PDB ; 2XB9,^29^ which has the inhibitor (2*R*)-2-(4-methoxybenzyl)-3-dehydroquinic acid in the active site, was employed. For *Mt*DHQ2, the reaction mechanism was studied in the forward (formation of **3**) and backward direction (formation of **1**). Because the latter provided more reliable results with less motion of the key residues (in line with efficient reaction), both enzymatic conversions are studied and compared in the direction of formation of **1** here. The ligand present in the PDB ; 2XB9 structure was replaced by the product, 3-dehydroshikimic acid (**3**). Except the structural water molecule (**W1**), all crystallographic waters were removed. The addition of missing hydrogen atoms and protonation states of residues were assigned using the H++ web server at pH 7.0 (; http://biophysics.cs.vt.edu/H++),^39^ except for Asp88′/Asp89′ that was treated as protonated due to mechanistic considerations. The ff03^40^ AMBER force field and GAFF^41^ were used to assign bonded and non-bonded parameters to the protein and both 3-dehydroquinic acid (**1**) and 3-dehydroshikimic acid (**3**), respectively. Each complex was immersed in a truncated octahedron of TIP3P^42^ water molecules and neutralised with Na^+^ ions^43^ (∼20 000 water molecules and 15 Na^+^ ions for the *Mt*DHQ2 model and ∼30 000 water molecules and 16 Na^+^ ions for the *Hp*DHQ2 model were added).

### Molecular dynamics simulations

Simulations were performed using the *sander* and *pmemd* modules of AMBER 12.^44^ Periodic boundary conditions were applied and electrostatic interactions were treated using the smooth PME (particle mesh Ewald) method^45^ with a grid spacing of 1 Å. The cut-off distance for the nonbonded interactions was 9 Å, the SHAKE^46^ algorithm was applied to all bonds and an integration step of 2.0 fs was used throughout. After an initial energy minimization of the water molecules and counter ions, both systems were heated to 300 K in 25 ps using positional restraints of 50.0 kcal mol^–1^ Å^–2^ for the protein Cα atoms. The *Hp*DHQ2 model was then equilibrated over 200 ps with the positional restraints, which were subsequently reduced by 5.0 kcal mol^–1^ Å^–2^ every 20 ps, followed by 20 ps without restraints. The *Mt*DHQ2 model (with coordinates from **3** already present in the crystal structure) was equilibrated for 100 ps using restraints of 5.0 kcal mol^–1^ Å^–2^ for the protein Cα atoms (except for amino acids Asp89′, Arg19 and Glu20). Subsequently, restraints were reduced by 1.0 kcal mol^–1^ Å^–2^ every 20 ps, and another 20 ps without restraints. To maintain a catalytically relevant conformation, a restraint of 100 kcal mol^–1^ Å^–2^ was used to keep the dihedrals CB, CG, OD2, HD2 of Asp88′ at 180° and CA, CB, CG, OD2 of Asp89′ between 120° and –60°. Two further one-sided distance restraints of 15 kcal mol^–1^ Å^–2^ were used: one to keep the CG_Asp89′_–CD_Glu20_ distance to within 4 Å and another to keep the CD_Glu20_–CZ_Arg108_ distance to at least 6.5 Å, in order to avoid distortion of the active site. For both models, the final equilibration snapshot was used for mechanistic studies with QM/MM MD.

### QM/MM MD simulations

For both enzymes, the QM region included the equivalent atoms of the product, 3-dehydroshikimic acid (**3**), the side chains of residues Arg19/Arg17, Tyr24/Tyr22, His101/His102 and Asp88′/Asp89′, and the product and structural (**W1**) and product (henceforth **W3**) water molecules. For the *Hp*DHQ2 model, a second water molecule, **W2**, that was observed located between Tyr22 and Asp89′ was also included. Hydrogen ‘link atoms’^47^,^48^ were used to model bonds across the QM/MM boundary, specifically between Cγ and Cδ of Arg19/Arg17, Cα and Cβ of Tyr24/Tyr22, His101/His102 and Asp88′/Asp89′. **W3** was manually docked in the carboxylate recognition pocket, establishing one hydrogen bond with the Oδ1 oxygen atom of Asn75/Asn76 and another one with the Nδ1 nitrogen atom of His101/His102. QM/MM calculations were performed using sander from AMBER 12 (version 12.19). SCC-DFTB^49^ was used for the QM region.

QM/MM umbrella sampling MD simulations were run for each reaction step, harmonically restraining the reaction coordinate with a force constant of 100 kcal mol^–1^ Å^–2^. Each simulation (window) consisted of 5 ps (10 000 steps with an integration step of 0.5 fs) of sampling and the reaction coordinate was increased (or decreased) by 0.1 Å between neighboring windows, using the last geometry of the previous window as starting point. Values of the reaction coordinate were collected for all simulation steps. The free-energy profiles for each step were obtained by combining the statistics from all simulations for that reaction using the weighted histogram analysis method (WHAM).^50^–^52^ The following reaction coordinates were used: (i) *third step* – for both enzymes, a 2D umbrella sampling simulation involving the formation of the C1–oxygen bond in **1**, and the proton transfer from **W3** to His101/His102 was used: *r*_1a_ = *d*(C1–O_W3_) and *r*_1b_ = *d*(ND1_His_–H1W_3_) – *d*(H1W_3_–O_W3_). (ii) *second step* – for both enzymes, a single reaction coordinate for the hydrogen addition to C2 in **1** by Tyr24/Tyr22 was used: *r*_2_ = *d*(HH_Tyr_–C2) – *d*(HH_Tyr_–HO_Tyr_). Moreover, several additional restraints were employed to avoid sampling rearrangements not related to the chemical steps of interest (*e.g.* ligand unbinding, see ESI† for details). (iii) *first step* – For the *Mt*DHQ2 model, a single reaction coordinate for protonation of the catalytic tyrosinate Tyr24 was employed: *r*_3a_ = *d*(OH_Tyr22_–HD2_Asp89′_) – *d*(HD2_Asp89′_–OD2_Asp89′_), and an additional reaction coordinate was needed for the approach of the neutral Asp89′ to the tyrosinate anion (see ESI† for details). For the *Hp*DHQ2 model, 2D umbrella sampling with two reaction coordinates was required: (1) deprotonation of **W2** by tyrosinate Tyr22, and (2) protonation of **W2** by neutral Asp88′, *r*_3a_ = *d*(OH_Tyr22_–H1W_2_) – *d*(H1W_2_–O_W2_) and *r*_3a_ = *d*(HD2_Asp88′_–O_W2_) – *d*(HD2_Asp88′_–OD2_Asp88′_).

### High level QM corrections and residue contributions for the 2nd step

For both enzymes, snapshots of the 0.0 Å window in the 2^nd^ step were minimized in a three step procedure: (1) 500 steps (20 steepest descent followed by 480 conjugate gradient) with the same restraints used in umbrella sampling but with a force constant of 2500 kcal mol^–1^ Å^–2^; (2) minimization using the LBFGS method (ntmin = 3) and a convergence criterion of 0.002 kcal mol^–1^ (drms = 0.002) keeping only restraints to the reaction coordinate; (3) minimization with positional restraints of 50 kcal mol^–1^ Å^–2^ to any residue, water molecule or ion that was more than 5.0 Å away from the QM region and the restraint of the reaction coordinate. The reaction coordinate (between –1.1 to 1.0 Å for *Mt*DHQ2 and –1.2 to 1.5 Å for *Hp*DHQ2) was explored forwards and backwards until a converged and smooth (adiabatic) potential energy profile along the reaction pathway was obtained. Coordinates for the QM region (with H link atoms) along the reaction pathway were then extracted and single point energy calculations in vacuum with Gaussian09^53^ with SCC-DFTB and MP2 (with the 6-31+G(d,p) basis set) were performed. SCS-MP2,^54^ B3LYP^55^,^56^ and MPW1K^57^ single point energy calculations were also performed. Corrections to the potential and free energy surfaces (PES and FES) were applied by subtracting the energy calculated for the QM region using SCC-DFTB and adding the energy from the MP2 (or other) calculations. Similar approaches have been applied successfully previously to other enzyme-catalyzed reactions;^58^,^59^ it corrects for limitations of the lower-level method *e.g.* in the calculation of proton affinities. Individual electrostatic contributions for residues Asp88′/89′ and Arg19/Arg17 and water molecules **W1** and **W2** (only *Hp*DHQ2) were then evaluated by calculating the difference between the energy profile with or without the corresponding residue present. These energies were calculated as single points with the same methods as before.

Due to the more complicated reaction coordinate for the 3^rd^ step (involving carbon–oxygen bond breaking as well as a proton transfer, which necessitates two-dimensional umbrella sampling) as well as relevant solvent rearrangements, a similar energy correction based on single-point energies from a potential energy profile is not reliable for this reaction step. Nevertheless, we performed such a correction to give an approximate indication of how the reaction is affected by more accurate QM treatment (Table S7†). The approximate corrections would lead to free-energy barriers for step 3 (relative to the reactant state) of ∼11.5 kcal mol^–1^ for *Mt*DHQ2 and ∼16.1 kcal mol^–1^ for *Hp*DHQ2. In cases such as this, simple potential energy profiles of enzyme reactions suffer from extreme variability due to conformation variability and solvation changes and are not an effective approach to modelling the mechanism. Single structures do not represent the ensemble of structures, and structural changes involved in reaction. Indeed, if this approach were applicable, we would have followed such an approach, as we have done in some previous work. It is not in this case, as we have established by test calculations. Instead, it is essential to sample conformational changes and generate appropriate structural ensembles for the reacting system through QM/MM molecular dynamics simulations. This necessitates the use of a low-level QM/MM method, in this case SCC-DFTB, with corrections where necessary (see below). We have discussed these factors (and approaches to modelling enzyme reactions in general) in ref. 11 and 60–62 as well as in a large number of published applications (ref. 12 and 63).

Specific errors for sampling can be obtained by repeating the reaction simulations multiple times. Where we have done this in the past using a similar approach (and known enzyme–substrate complexes), we find standard deviations of 0.1–1 kcal mol^–1^.^12^

## Conflicts of interest

There are no conflicts to declare.

## Abbreviations

DHQ2: Type II dehydroquinase
*Mt*DHQ2: Type II dehydroquinase from *Mycobacterium tuberculosis*
*Hp*DHQ2: Type II dehydroquinase from *Helicobacter pylori*

## Supplementary Material

Supplementary information
