# Supplementary material for: QM/MM simulations identify the determinants of catalytic activity differences between type II dehydroquinase enzymes
Source: Org Biomol Chem. 2018 May 16;16(24):4443–55. doi: 10.1039/c8ob00066b (PMC6011038; doi:10.1039/c8ob00066b)
Supplement: Supplementary file 1 [file OB-016-C8OB00066B-s001.pdf]

Electronic Supplementary Material (ESI) for Organic & Biomolecular Chemistry.
This journal is © The Royal Society of Chemistry 2018

Supporting Information for:

QM/MM Simulations Identify the Determinants of Catalytic Activity Differences Between Type II Dehydroquinase Enzymes

Emilio Lence, Marc W. van der Kamp, Concepción González-Bello and Adrian J. Mulholland

Table of Contents

1. **Figure S1**………..………………..……………………….…………………………………...……. S2
2. **Figure S2**………..………………..……………………….…………………………………...……. S3
3. **Figure S3**………..………………..……………………….…………………………………...……. S4
4. **Figure S4**………..………………..……………………….…………………………………...……. S5
5. **Figure S5**………..………………..……………………….…………………………………...……. S6
6. **Figure S6**………..………………..……………………….…………………………………...……. S7
7. **Table S1**………..………………..……………………….…………………………………...…..…. S8
8. **Table S2**………..………………..……………………….…………………………………...…..…. S9
9. **Table S3**………..………………..……………………….…………………………………...…..…. S10
10. Reaction coordinates for Umbrella Samplings simulations with restraints……………………. S11
11. **Table S4**..……..………………..……………………….…………………………………...…..…. S12
12. **Figure S6**.……..………………..……………………….…………………………………...…..…. S14
13. **Table S5**.……..………………..……………………….…………………………………...…..…. S13
14. **Table S6**.……..………………..……………………….…………………………………...…..…. S14
15. **Table S7**.……..………………..……………………….…………………………………...…..…. S15

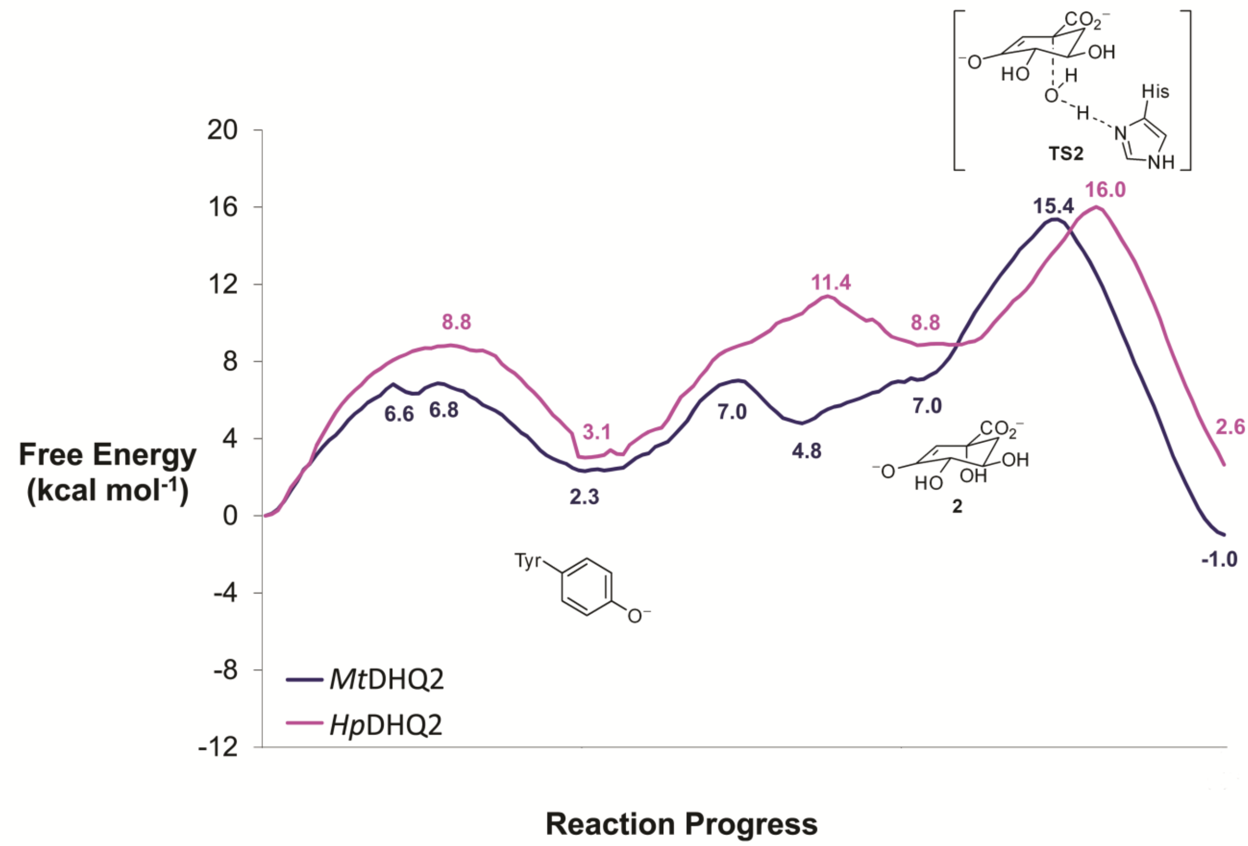

Figure S1. Free energy profiles obtained using Umbrella Sampling simulations at the SCC-DFTB/ff03 level for the whole reaction for both enzymes [*Mt*DHQ2 (blue), *Hp*DHQ2 (magenta)]. For 1st step in *Hp*DHQ2 and 3rd step in both enzymes, minimum free energy paths extracted from two-dimensional free energy surfaces are shown.

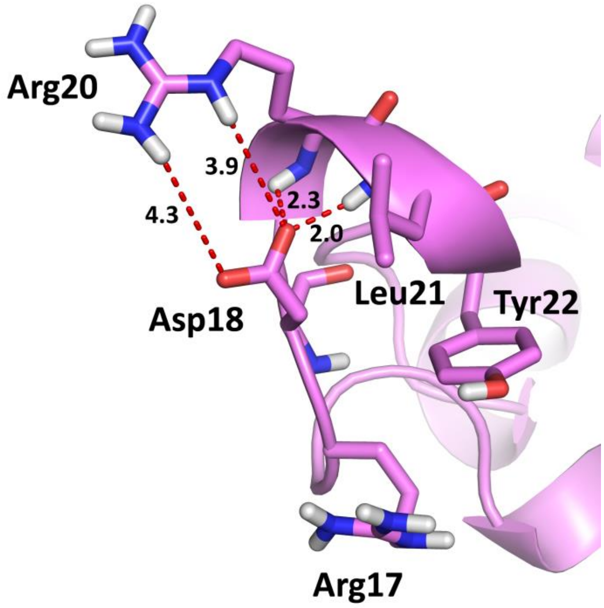

Figure S2. Detail view of the salt bridge between Arg20 and Asp18 in *Hp*DHQ2 enzyme observed during MD simulation studies. Hydrogen bonding interactions involving residues 18–21 are also highlighted. Relevant side chain residues are shown and labeled.

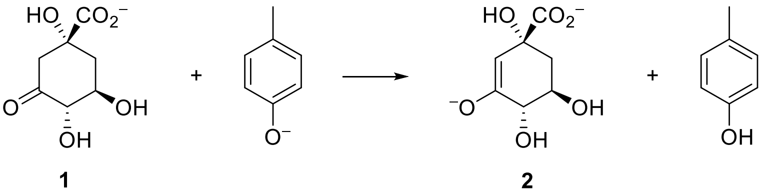

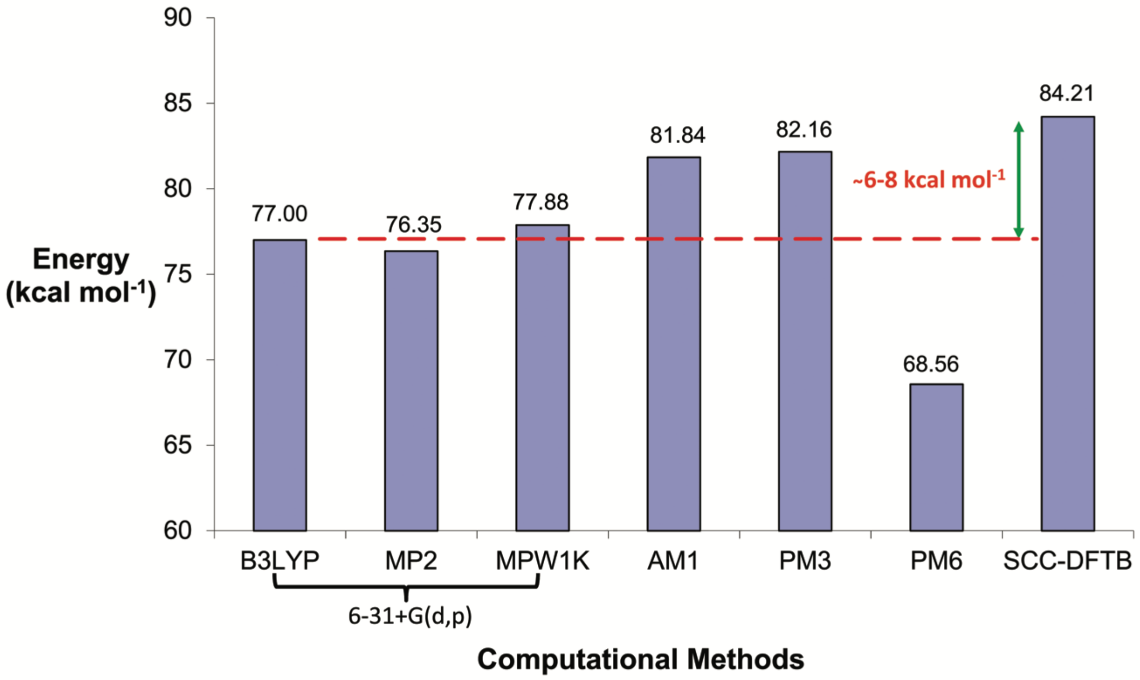

Figure S3. Comparison of reaction energies for the formation of the enolate **2** from substrate **1** with phenolate as model for Tyr24/Tyr22 as the base. Energies were obtained by single point calculations in vacuum using several levels of theory. SCC-DFTB provides energy values for the reaction that are about 6−8 kcal mol^{-1} higher than more accurate methods such as MP2, B3LYP or MPW1K.

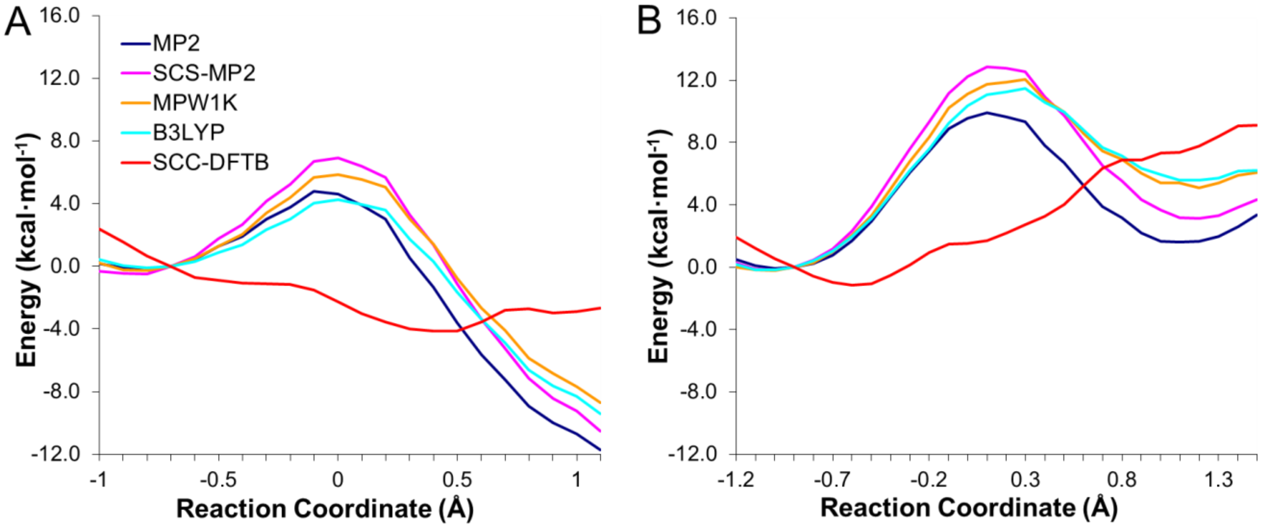

Figure S4. QM/MM potential energy profiles for the second reaction step in (A) *Mt*DHQ2 and (B) *Hp*DHQ2. Structures were optimized at the SCC-DFTB/ff03 QM/MM level, with energies at other QM levels obtained as single point energy corrections of the QM region (as described in the Methods section). The reaction coordinate is defined as the difference of the proton acceptor and donor distances: $d(OH_{Tyr24/22}\text{-}H) - d(C2_2\text{-}H)$.

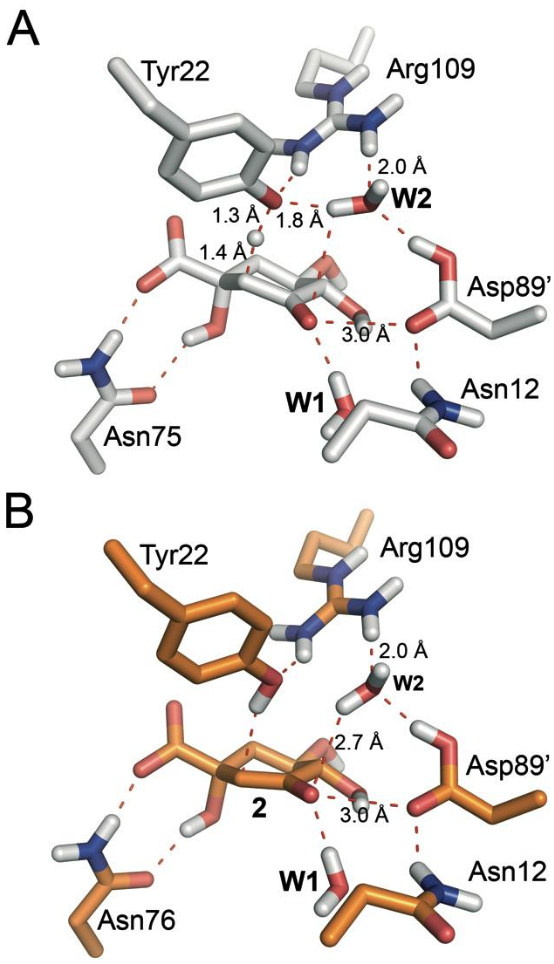

Figure S5. Detailed view of **TS1** (A) and enolate intermediate **2** (B) for the 2nd step of the *Hp*DHQ2 mechanism. Geometries were taken from the potential energy surface (SCC-DFTB/ff03 QM/MM level, see Figure S4). Relevant residues and water molecules are shown and labeled. Key hydrogen bonding interactions and bonds broken/formed are indicated as red dashed lines. Note how the catalytic water molecule **W2** interacts by hydrogen bonding with the neutral Asp89´ in both **TS1** and enolate intermediate **2**.

A

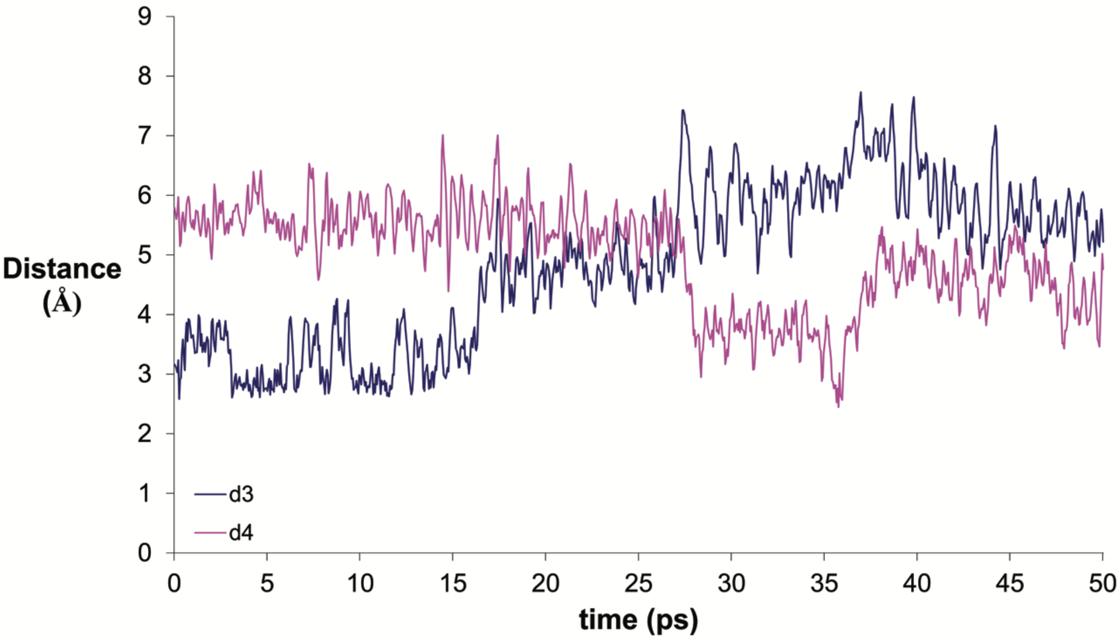

B

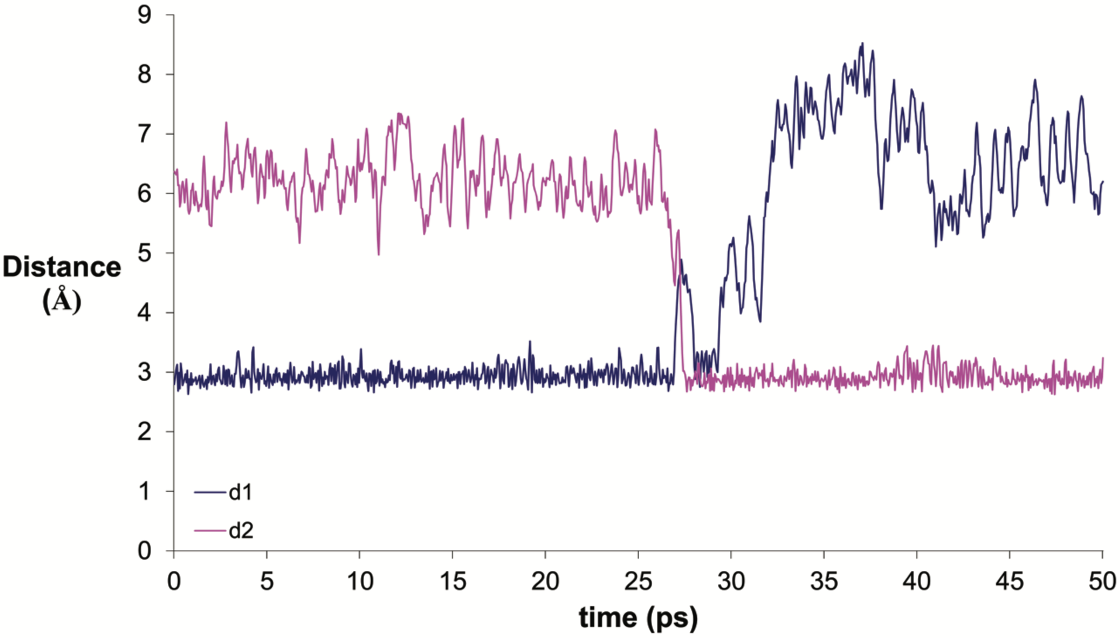

Figure S6. Variation of the distance between the oxygen atom of the water molecule **W2** and O3 atom in the enolate intermediate **2** (A) or the NH2 atom of Arg109 (B) during a 50 ps QM/MM simulation of the *Hp*DHQ2/**2** enzyme complex. Note how **W2** (from QM region) is replaced by another water molecule from the bulk solvent (from MM region) during the simulation. The distances were calculated considering the position of **W2** observed at the beginning of the simulation (blue trace) and of any water molecule that replaces **W2** during the simulation (magenta trace).

Table S1. Contributions of residues Asp88´ and Arg17 and water molecules **W1** and **W2** to the stabilisation of **TS1** and enolate intermediate **2** for the reaction catalysed by *Hp*DHQ2 at several levels of theory (kcal mol^{-1}) relative to substrate and tyrosinate states.[a]

Method	Residue/water molecule	**TS1**	**2**
MP2	**W2**[b]	−8.60	−16.89
SCS-MP2	**W2**	−8.33	−16.47
DFTB	**W2**	−8.01	−14.62
MPW1K	**W2**	−9.19	−17.94
B3LYP	**W2**	−9.16	−17.87
RHF	**W2**	−7.70	−16.03
$MPW1K_{wat}$	**W2**	−5.50	−10.64
MP2	Asp88’[c]	−2.07	−4.56
SCS-MP2	Asp88’	−1.95	−2.45
DFTB	Asp88’	−1.49	−3.95
MPW1K	Asp88’	−2.15	−4.49
B3LYP	Asp88’	−2.37	−4.54
RHF	Asp88’	−1.77	−3.64
$MPW1K_{wat}$	Asp88’	−1.28	−2.62
MP2	Arg17[d]	4.30	15.21
SCS-MP2	Arg17	4.08	15.59
DFTB	Arg17	2.69	11.14
MPW1K	Arg17	4.23	15.10
B3LYP	Arg17	4.25	14.17
RHF	Arg17	2.85	13.46
$MPW1K_{wat}$	Arg17	3.51	8.24
MP2	**W1**[e]	0.89	2.89
SCS-MP2	**W1**	0.76	2.78
DFTB	**W1**	0.96	2.25
MPW1K	**W1**	0.60	2.07
B3LYP	**W1**	0.52	1.73
RHF	**W1**	0.07	1.46
$MPW1K_{wat}$	**W1**	0.60	2.53

[a]The geometries for the substrate **1**, the enolate intermediate **2** and **TS1** were extracted from the QM region. Single point energies in vacuum at the corresponding theory method and 6-31+G(d,p) as basis set (when needed), were calculated for: (a) the full QM region; (b) the QM region without **W2**; (c) QM region without **W2** and Asp88’; (d) QM region without **W2** and Arg17; and (e) QM region without **W2** and **W1**. For $MPW1K_{wat}$, the energy was calculated in water as implicit solvent (Polarizable continuum model with default settings). All energies are relative to the energy for the substrate **1** (i.e. equivalent energy calculated for the substrate geometry was set to 0 kcal mol^{-1}). [b]Energy contribution for **W2** was calculated by subtracting the energy of (a) from (b). [c]Energy contribution for Asp88´ was calculated by subtracting the energy of (b) from (c). [d]Energy contribution for Arg17 was calculated by subtracting the energy of (b) from (d). [e]Energy contribution for **W1** was calculated by subtracting the energy of (b) from (e). Note that positive values indicate a higher stabilization effect on **2** relative to **1**, while negative values indicate the opposite.

Table S2. Contributions of residues Asp89´ and Arg19 and water molecule **W1** to the stabilisation of **TS1** and enolate intermediate **2** for the reaction catalysed by *Mt*DHQ2 at several levels of theory (kcal mol^{-1}) relative to substrate and tyrosinate states.[a]

Method	Residue/water molecule	**TS1**	**2**
MP2	Asp89'[b]	1.51	1.67
SCS-MP2	Asp89'	1.52	1.60
DFTB	Asp89'	0.95	0.91
MPW1K	Asp89'	1.78	1.93
B3LYP	Asp89'	1.97	1.77
RHF	Asp89'	1.94	1.86
$MPW1K_{wat}$	Asp89'	1.03	1.10
MP2	Arg19[c]	−2.74	6.20
SCS-MP2	Arg19	−2.53	6.52
DFTB	Arg19	−0.30	2.75
MPW1K	Arg19	−2.49	6.42
B3LYP	Arg19	−1.94	6.41
RHF	Arg19	−1.87	6.60
$MPW1K_{wat}$	Arg19	0.47	6.49
MP2	**W1**[d]	1.13	4.03
SCS-MP2	**W1**	1.30	3.96
DFTB	**W1**	1.19	3.52
MPW1K	**W1**	0.88	3.09
B3LYP	**W1**	0.80	2.74
RHF	**W1**	0.40	2.63
$MPW1K_{wat}$	**W1**	0.86	3.28

[a]The geometries for the substrate **1**, the enolate intermediate **2** and **TS1** were extracted from the QM region. Single point energies in vacuum at the corresponding theory method and 6-31+G(d,p) as basis set (when needed), were calculated for: (a) the full QM region; (b) the QM region without Asp89'; (c) the QM region without Arg19; (d) QM region without **W1**. For $MPW1K_{wat}$, these energies were calculated in water as implicit solvent (Polarizable continuum model with default settings). All energies are relative to the energy of the substrate **1**. [b]Energy contribution for Asp89' was calculated by subtracting the energy of (a) from (b). [c]Energy contribution for Arg19 was calculated by subtracting the energy of (a) from (c). [d]Energy contribution for **W1** was calculated by subtracting the energy of (a) from (d). Note that positive values indicate a higher stabilization effect on **2** relative to **1**, while negative values indicate the opposite.

Table S3. Estimation of the strength (kcal mol^{-1}) of the hydrogen bond between a water molecule and *p*-methylphenol and *p*-methylphenolate as mimics of tyrosine and tyrosinate residues, respectively, and the O3 atom in **1** and **2**.

Interaction	MPW1K SP[a]	MPW1K opt[b]
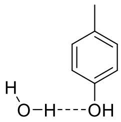	−0.25	−4.51
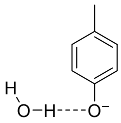	−19.36	−17.73
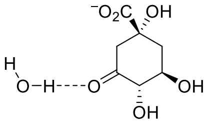 **1**	0.44	−13.91
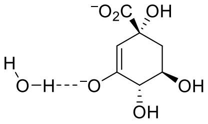 **2**	5.05	−21.53

[a]The geometries for the studied species were extracted from the potential energy surface of the 2nd step of the conversion catalysed by the *H. pylori* enzyme (Figure S4). Energy values were calculated in vacuum using MPW1K/6-31+G(d,p) as a difference of the energy for both species together minus the sum of the energies for each one alone. [b]Geometries for the hydrogen bond were optimized at the same level of theory as before. Note how the hydrogen bond between a water molecule and phenolate is stronger than with the corresponding neutral phenol. A smaller difference was found for the respective interaction with an enolate *vs* a ketone group.

Reaction coordinates for Umbrella Samplings simulations with restraints

(A) *Mt*DHQ2 model

The reaction coordinates and the restraints included in each step are summarized in Table S4. Because the reaction mechanism was evaluated in the backwards direction, starting from the product complex, the reaction steps are described below in that order (third, second and first step).

(i) For the third step, two reaction coordinates were used: r_{1a} that is the distance between C1 atom in **3** and the O atom of the manually docked product water molecule and r_{1b} represents the deprotonation of the water molecule by His101 residue. A 1D Umbrella Sampling simulation along reaction coordinate r_{1a} decreasing distance from 3.5 to 1.5 Å was initially performed, however only the results of the WHAM analysis between 2.3 and 3.5 Å were considered due to poor overlapping of the frecuency histograms of the reaction coordinate in the windows 1.7 to 2.0 Å. In order to achieve a better description of the formation of the C1−O bond as well as the water deprotonation, a 2D Umbrella Sampling was carried out between the distance values of 1.5 and 2.3 for r_{1a} and 1.0 to −1.0 Å for r_{1b} using the restraints included in Table S4. Restraint M30b was used to prevent the collapse of the active site caused by the attraction between Arg108 and Glu20 (it was previously used during equilibration). A01 was employed to avoid deprotonation of the water molecule and subsequent undesired side reactions. A02m was utilised to keep Tyr24 in the active site. A03 was used to prevent spontaneous protonation of C2 once the enolate intermediate **2** was formed. Finally, restraints A04a and A04b were applied to avoid formation of an enol intermediate instead of enolate **2**. The final structure of the window for 1.5 Å and −0.8 Å was selected as a suitable starting point for the 2nd step.

(ii) For the second step, a single reaction coordinate (r_2), which represents the proton transfer from Tyr24 to C2 in **2**, was used and evaluated from 1.1 to −1.0 Å using the restraints indicated in Table S4. Restraints A05 and M06 were employed to avoid the protonation of the catalytic tyrosinate by Arg19 and the subsequent protonation of the resulting neutral guanidinium by Asp89’. Restraints M04 and M05 were used to ensure the correct binding of C1 region of the natural substrate. The final structure of the window for −0.8 Å was selected as a suitable starting point for the 1st step.

(iii) For the first step, two reaction coordinates were employed: r_{3a} that is a linear combination of distances to break the Glu20−Asp89’ interaction and r_{3b} that corresponds to the deprotonation of neutral Asp89’ by the catalytic tyrosinate. Using the restraints indicated in Table S4, reaction coordinate r_{3a} from −1.9 to 1.8 Å was evaluated. The final structure of the window for −0.1 Å was selected as a suitable starting point for r_{3b}. Reaction coordinate r_{3b} from −0.6 to 1.0 Å was then

followed. Restraints M12 and M09 were applied to prevent the regeneration of the enolate intermediate **2** and to avoid the formation of an enol intermediate, respectively. Restraints M07 and M08 were employed to keep Arg19 in the active site. Restraint M10 was used to prevent displacement of the water molecule **W1** from its binding pocket. Finally, restraints M11a and M11b were included to ensure that Glu20 was not expelled from the active site.

Table S4. Reaction coordinates and the restraints for *Mt*DHQ2 model.

**Step**	**Reaction Coordinates**[a]	**Restraints**[b]
3rd	r_{1a} = d(C1**3** – O_W)<br>r_{1b} = d($ND1_{His101}$ – $H1_W$) – d($H1_W$ – O_W)	A01, A02m, A03, A04a, A04b, M03b
2nd	r_2 = d(C2**2** – HH_{Tyr24}) – d(HH_{Tyr24} – OH_{Tyr24})	A05, M03b, M04, M05, M06
1st	r_{3a} = d(CD_{Glu20} – $CG_{D89'}$) – d($CG_{D89'}$ – CZ_{Tyr22})	A04a, A04b, M03b, M04, M05, M07, M08, M09, M10, M11a, M11b, M12
	r_{3b} = d(OH_{Tyr24} – $HD2_{Asp89'}$) – d($HD2_{Asp89'}$ – $OD2_{Asp89'}$)	M03b, M04, M05, M08

[a]See Figure S7 for more details. [b]The type of restraint, the force constant and the atoms involved are indicated in Table S6.

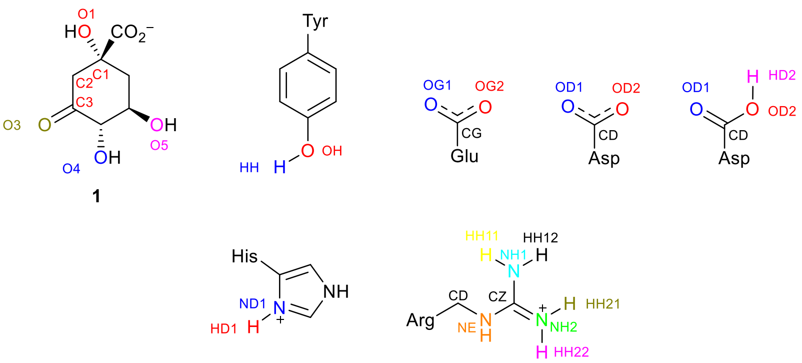

Figure S7. Schematic representation of the atom names used in the definition of the reaction coordinates (Tables S4-S5) and restraints (Table S6).

(B) *Hp*DHQ2 model

The reaction coordinates and the restraints included in each step are summarized in Table S5.

(i) For the third step, a similar procedure as for the *Mt*DHQ2 model was used. Only the particular differences are explained. Thus, the r_{1b} reaction coordinate was evaluated from 1.0 to −0.6 Å using the restraints indicated in Table S5. The final structure of the window for 1.5 Å and −0.6 Å was selected as a suitable starting point for the 2nd step. Restraints A02h and H03 were used to keep the Tyr24 in the active site and to avoid formation of an enol instead of enolate **2**, respectively. Restraints H01a and H02a were applied to avoid diffusion of the water molecule **W2** into the bulk solvent. Restraints H04, H05 and H06 were introduced to maintain Arg17 in the active site.

(ii) For the second step, as for *Mt*DHQ2 model a single reaction coordinate r_2 was employed. r_2 was evaluated from 1.5 to −1.2 Å using the restraints shown in Table S5. The final structure of the window for −0.9 Å was selected as a suitable starting point for the 1st step. Restraint A05 was employed needed to circumvent the formation of an enol instead of the enolate **2**. Restraints H01b and H02b were applied to avoid diffusion of the water **W2** into the bulk solvent.

(iii) For the first step, two reaction coordinates were employed: r_{3a} that is the deprotonation of the catalytic water molecule **W2** by the catalytic tyrosinate and r_{3b} that involves the abstraction by **W2** of the proton from the neutral Asp88'. In both cases the reaction coordinate varied from 0.8 to −0.8 Å and no additional restraints were applied.

Table S5. Reaction coordinates and the restraints for *Hp*DHQ2 model.

**Step**	**Reaction Coordinates**[a]	**Restraints**[a]
3rd	$r_{1a} = d(C1_{\mathbf{3}} – O_W)$<br>$r_{1b} = d(ND1_{His101} – H1_W) – d(H1_W – O_W)$	A01, A02h, A03, A04a, A04b, H01a, H02a, H03, H04, H05, H06
2nd	$r_2 = d(C2_{\mathbf{2}} – HH_{Tyr22}) – d(HH_{Tyr22} – OH_{Tyr22})$	A04a, A04b, A05, H01b, H02b, H05
1st	$r_{3a} = d(OH_{Tyr22} – H1_{\mathbf{W2}}) – d(H1_{\mathbf{W2}} – O_{\mathbf{W2}})$<br>$r_{3a} = d(O_{\mathbf{W2}} – HD2_{Asp88'}) – d(HD2_{\mathbf{Asp88'}} – OD2_{\mathbf{Asp88'}})$	---

[a]See Figure S7 for more details. [b]The type of restraint, the force constant and the atoms involved are indicated in Table S6.

Table S6. Restraints used during the Umbrella Sampling simulations

Code	Type	Atoms involved[a]	Value[b]	Force constant[c]
M01	Dihedral	(D89’) CB–CG–OD2–HD2	180	100
M02	Distance	(D89’) CG – CD (E18)	< 4.0	15
M03a	Distance	(E18) CD – CZ (R108)	> 6.5	15
M03b	Distance	(E18) CD – CZ (R108)	> 7.0	15
A01	Distance	(W) O – H2 (W)	< 1.1	100
A02m	Distance	(Y22) OH – C2 (**3**)	< 3.0	15
A02h	Distance	(Y24) OH – C2 (**3**)	< 3.0	25
A03	LCOD[d]	d[Y22/Y24(OH-HH)] – d[Y22/Y24 (HH)2-C2 (**3**)]	< −1.0	100
A04a	Distance	R17/R19 (NH2 – HH22)	< 1.1	100
A04b	Distance	R17/R19 (NH2 – HH21)	< 1.1	100
H01a	Distance	(Y24) OH – O (W2)	< 4.0	25
H01b	Distance	(Y24) OH – O (W2)	< 3.0	25
H02a	Distance	(D88’) CG – O (W2)	< 4.5	25
H02b	Distance	(D88’) OD2 – O (W2)	< 3.0	25
H03	LCOD	d[D88’(OD2-HD2)] – d[D88’(HD2)-O3 (**1**)]	< −1.0	100
H04	Distance	(R19) NH1 – O3 (**3**)	< 3.3	25
H05	Distance	(R19) NH2 – OD1 (D89’)	< 3.3	25
H06	Angle	(R19) NH1 –HH12 (R19) – OD1 (D89’)	180	25
M04	Distance	(**3**) O1 – ND1 (H101)	< 3.5	100
M05	Angle	(**3**) O1 – HD1 (W) – ND1 (H101)	180	100
A05	Distance	(D89’/D88′) OD2 – HD2 (D89′/D88’)	< 1.1	100
M06	Distance	(R17) NH2 – HH22 (R17)	< 1.1	100
M07	Distance	(R17) NH2 – O3 (**3**)	< 4.0	100
M08	Angle	(R19) NH2 – R (HH22) – O3 (**3**)	> 150	25
M09	LCOD	d[R19(NH2-HH2)] – d[(R19) HH22 − O3 (**3**)]	< −1.0	100
M10	Distance	(W1) O – O3 (**1**)	< 4.0	100
M11a	Distance	(W3) O – CD (E20)	< 4.0	100
M11b	Distance	(W3) O – CZ (R108)	< 4.0	100
M12	LCOD	d [(**3**) C2 – HH (Y24)] – d [(Y24) HH – OH (Y24)]	< -1.0	100

[a]See Figure S7 for more details. [b]Distance in Å, angles in degrees. [c]Units (kcal mol^{-1} $Å^{-2}$) or (kcal mol^{-1} rad^{-2}). [d]LCOD is Linear Combination of Distances.

Table S7. Energy differences (kcal mol^{-1}) for the formation of the reaction product from enolate **2** (step 3). Energies were obtained by single point calculations in vacuum using several levels of theory based on structures extracted from the minima and TS maximum of a two dimensional SCC-DFTB/MM potential energy profile.

Enzyme	Level of theory	TS	Products
*Mt*DHQ2			
	MP2	+ 4.3	+ 14.6
	B3LYP	+ 2.7	+ 10.6
	MPW1K	+ 5.3	+ 15.3
*Hp*DHQ2			
	MP2	+ 5.8	+ 9.6
	B3LYP	+ 2.6	+ 5.9
	MPW1K	+ 7.7	+ 2.8
